# Color-Tunable Intrinsically Black Polyimides: A Facile Strategy via In Situ Oxidation Color Control

**DOI:** 10.3390/polym17212876

**Published:** 2025-10-28

**Authors:** Desheng Kong, Jiaojiao Ma, Zeyu Li, Shun Shi, Tong Yuan, Jianfeng Qian, Haiquan Guo

**Affiliations:** 1 Chang Chun Institute of Applied Chemistry Chinese Academy of Sciences Changchun, No. 5625, Renmin Avenue, Changchun 130022, China; kongdesheng@ciac.ac.cn (D.K.); zyli2@ciac.ac.cn (Z.L.); shishun@ciac.ac.cn (S.S.); yunhuichen@ciac.ac.cn (T.Y.); trushz@163.com (J.Q.); 2 School of Applied Chemistry and Engineering, University of Science and Technology of China, Hefei 230026, China

**Keywords:** intrinsically black polyimide, in situ oxidation, color control, diphenylamine

## Abstract

Black polyimide (BPI) has shown important value in the field of optical engineering due to its excellent light shielding, high temperature stability, and mechanical strength. However, carbon materials or organic dye-doped BPI suffer from poor insulation, low mechanical strength, and poor thermal stability. Intrinsic BPI has gradually become a hot topic of research at this stage. Polyimides containing dianiline structure have unique reducing activity, and the visible light absorption range can be expanded by adding an oxidant in situ to achieve BPI preparation. In this work, a polymerizable dianiline derivative- 2,4-diaminodiphenylamine (NPDA) has been developed. The resulting diamine monomers were then polymerized with a dianhydride monomer via a conventional two-step method to prepare soluble polyimide. The diphenylamine-containing group PI was characterized by 1H NMR, FTIR and UV absorption spectroscopy. It was found that by changing the oxidant ratio, a yellow, red and even black controllable polyimide film could be obtained. When fully oxidized, the BPI cutoff wavelength red shifts to 591 nm, light transmittance reaches as low as 5.9% (full visible light 300–700 nm mean), and BPI can maintain the electrical insulation and heat resistance of polyimide. This method of oxidizing soluble polyimide in situ has advantages such as economy, universality, process consistency, ease of access and superior performance.

## 1. Introduction

Polyimides are high-performance polymers widely used in advanced technologies due to their excellent thermal stability, mechanical strength, and dielectric properties [1,2,3]. However, commercial all-aromatic polyimide films often appear yellow to dark brown because of charge transfer complex (CTC) formation [4,5]. To address this, research has focused on modulating CTCs to develop colorless [6,7], colored [8,9], and black [5,10] polyimide films. Among these, black polyimide (BPI) films have gained attention for their strong visible-light absorption, making them suitable for applications such as protective layers in lithium-ion batteries [11], and coverlays in flexible printed circuit boards [12,13].

BPI films are valuable in optical engineering due to their high absorbance, thermal stability, and mechanical strength. They are used in satellite optics, camera flashes, automotive headlights, and optical attenuators, with recent aerogel-based BPIs achieving >99.7% absorbance and maintaining thermal stability up to 600 °C [14]. In microelectronics, BPI serves as a protective layer in integrated circuits and as a light-blocking layer in flexible OLED displays.

Current methods for producing BPI films include surface coating with black pigments, incorporation of black dyes, and intrinsic black polyimide synthesis via molecular design. Coating methods are straightforward but suffer from adhesion issues, while additive-based approaches often compromise electrical insulation or mechanical properties due to filler agglomeration [15,16,17,18,19,20,21,22]. Organic dyes offer better compatibility but lack thermal stability [4,23]. Intrinsic BPI films, synthesized through molecular design, as exemplified by monomers such as 4,4′-diaminodiphenylamine (NDA) [5,24], 2,4,5,7-tetraamino-1,8-dihydroxyanthraquinone (4NADA) [25,26], tetraphenylcyclopenta-dienone (TPCP) [12,27,28,29,30], and 3,6-bis(thiophen-2-yl)diketopyrrolopyrrole (TDPP) [31], provide excellent optical properties but face challenges in monomer synthesis, purification, and material performance.

In this work, we developed an in situ oxidation method during high-temperature polyimide film formation, enabling controllable color modulation from yellow through red to dark brown. A key aspect of our molecular design was the strategic introduction of oxidizable groups into the side chain of the polyimide, distinguishing our approach from prior studies that incorporated amino groups within the polymer backbone. We designed polyimides incorporating diphenylamine groups as side chains and regulated their coloration by introducing varying amounts of oxidizing agents during high-temperature film processing. 2,4-Diaminodiphenylamine (NPDA)—a key intermediate for synthesizing sulfur dyes (notably black/blue variants) and oxazine dyes—was employed as the diamine monomer to provide an oxidizable diphenylamine moiety as a side group rather than as part of the main chain. It was polymerized with 4,4′-oxydiphthalic anhydride (ODPA) to yield soluble polyimide precursors. Upon adding different types and concentrations of oxidants to this polyimide solution, we observed systematic color variations in the films during high-temperature curing. This phenomenon likely arises from the progressive oxidation of the amine groups in the diphenylamine side units. We systematically investigated the effects of reaction temperature, oxidant type, and concentration on the optical properties of the resulting PI films, elucidating the underlying color-change mechanism. Ultimately, precise control over oxidant content achieved tunable polyimide coloration from yellow to red and finally near-black. The in situ oxidized polyimide (BPI) exhibits a cutoff wavelength near 600 nm and maintains ultra-low visible-light absorption (≤5% across the spectrum). This side-chain oxidation strategy not only enables efficient, versatile, and cost-effective production of intrinsically black polyimide but also largely preserves the material’s inherent properties by maintaining the integrity of the polyimide backbone. Consequently, it represents a highly promising technical route for fabricating intrinsically black polyimide films.

## 2. Experimental Section

### 2.1. Materials

All commercially available materials were used as supplied without further purification unless otherwise stated. 4,4′-Oxydiphthalic anhydride (ODPA) was purchased from ChinaTech Chemical Co., Ltd. (Tianjin, China) and sublimated prior to use. Pd/C and 3-chloroperoxybenzoic acid (mCPBA) were obtained from InnoChem (Beijing, China), while 2,4-dinitrodiphenylamine was sourced from Adamas (Shanghai, China). N,N-Dimethylacetamide (DMAc), ethanol (EtOH), and tetrahydrofuran (THF) were acquired from General-Reagent (Shanghai, China) and Xilong Science (Shantou China), respectively.

### 2.2. Synthesis of 2,4-Dinitrodiphenylamine

A mixture of 1-bromo-2,4-dinitrobenzene (10.0 g, 40.4 mmol), aniline (4.1 mL, 44.4 mmol), and N, N-diisopropylethylamine (DIPEA, 7.8 mL, 44.4 mmol) was heated at reflux for 2 h (monitored by TLC). The resulting suspension was filtered, and the collected solid was dried under vacuum at 40 °C to afford 2,4-dinitrodiphenylamine (9.21 g, 88%) as an orange-red solid. ^1^H NMR(400 MHz, DMSO-*d*_6_, δ): 10.15 (s, 1H), 8.90 (d, J = 2.7 Hz, 1H), 8.23(dd, J = 9.5, 2.7 Hz, 1H), 7.61–7.47 (m, 2H), 7.38 (dd, 3H), 7.11 (d, J = 9.6 Hz, 1H).

### 2.3. Synthesis of 2,4-Diaminodiphenylamine

A mixture of 2,4-dinitrodiphenylamine (10.0 g, 38.6 mmol) and 10% Pd/C (3 mol%) in EtOH/THF (120 mL, *v*:*v* = 1:1) was treated with hydrazine hydrate (8 equiv) and stirred at room temperature or reflux. After 2 h (monitored by TLC), the reaction mixture was filtered through a bed of celite. The filtrate was concentrated under reduced pressure at 40 °C to remove THF, and the residue was precipitated in n-hexane. The resulting solid was collected by filtration, dried in vacuo over P_2_O_5_, yielding 2,4-diaminodiphenylamine (5.94 g, 77%) as a pale yellow solid. ^1^H NMR(400 MHz, DMSO-*d*_6_, δ): 7.09–6.94 (t, 2H), 6.72 (s, 1H), 6.61(d, J = 8.2 Hz, 1H), 6.56–6.42 (m, 3H), 6.00 (d, J = 2.4 Hz, 1H), 5.84 (dd, J = 8.2, 2.5 Hz, 1H), 4.65 (s, 2H), 4.43 (s, 2H).

### 2.4. Synthesis of PI

Polyimides were synthesized via chemical imidization in DMAc at room temperature, maintaining ≈ 20 wt% solid content and a 1:1 stoichiometric ratio of dianhydride to diamine. The room-temperature polymerization procedure was as follows: 2,4-Diaminodiphenylamine (2.0 g, 10.04 mmol) was dissolved in DMAc (20.44 g), followed by addition of ODPA (3.11 g, 10.04 mmol) with stirring at room temperature for 12 h. Subsequently, a mixture of triethylamine (2 equiv) and acetic anhydride (4 equiv) was added dropwise, and stirring continued for an additional 12 h. The resulting PI solution was quenched in water, filtered, and the collected solid was dried under vacuum to yield light yellow PI resin.

### 2.5. Preparation of PI Films

The PI resin was redissolved in DMAc (20 wt%), followed by addition of varying molar equivalents of oxidant mCPBA (0–100% in 20% increments). After stirring for 1 h at room temperature (or specified oxidation temperatures), the solution was filtered through a 5 μm glass fiber membrane and coated uniformly onto glass substrates. Films were initially baked at 70 °C for 0.5 h for preliminary setting, then cured at 150 °C for 1 h for oxidation, and finally post-cured at 250 °C for 1 h to complete imidization. After cooling, the film was demolded by water immersion.

### 2.6. Characterization

FTIR spectra of PI films were obtained by accumulating 128 scans on an INVENIO-R FTIR spectrometer (Bruker, Ettlingen, Germany) in attenuated total reflection (ATR) mode over the range of 4000–400 cm^−1^. ^1^H NMR spectra were recorded on a Bruker Avance 300 Spectrometer (Bruker Optic GmbH, Ettlingen, Germany). The residual solvent peak was used as an internal reference (DMSO-*d*_6_: δ_H_ = 2.50 ppm; CDCl_3_: δ_H_ = 7.26 ppm). Optical transmission spectra of 25 μm thick PI films were recorded on a UV-1900i spectrophotometer (Shimadzu, Kyoto, Japan) at room temperature over the spectral range of 300–700 nm. CIE L*A*B* values of the 25 μm BPI specimens were measured by YS6060 Benchtop Grating Spectrophotometer (3nh, Shenzhen, China) with CIE standard illuminant D65 and a 10° field of view. Film samples (50 mm × 10 mm × 0.025 mm) were strained at 5 mm/min on a Instron-1121 universal electromechanical tester (Instron Engineering Corporation, Boston, MA, USA) to record their mechanical properties. The decomposition behavior of PI films was monitored by thermogravimetric analysis (TGA) using a PerkinElmer 8000^TM^ (PerkinElmer, Hopkinton, MA, USA) under nitrogen at a heating rate of 10 °C min^−1^. Dynamic mechanical analysis (DMA) of BPI films was performed on a DMA (Rheometric Scientific Inc., New Castle, DE, USA) at a heating rate of 5 °C min^−1^ and a load frequency of 1 Hz under nitrogen. The coefficients of thermal expansions (CTEs) of the films were collected on a Q400 TMA (TA Instruments, New Castle, DE, USA) at a heating temperature rate of 5 °C min^−1^ in a nitrogen flow of 50 mL/min under a static load set at 0.05 N. The average CTE values from 50 to 200 °C were obtained from the second heating run after the first run, which was carried out to eliminate the residual stress.

## 3. Result and Discussion

### 3.1. Synthesis and Characterization of NPDA

**Figure 1:**
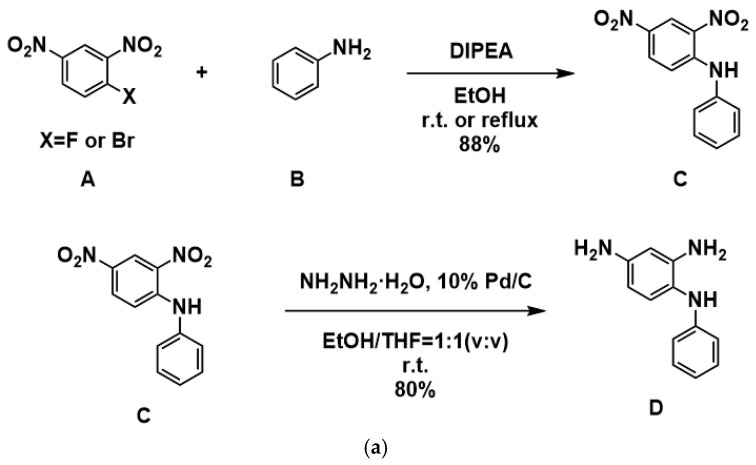

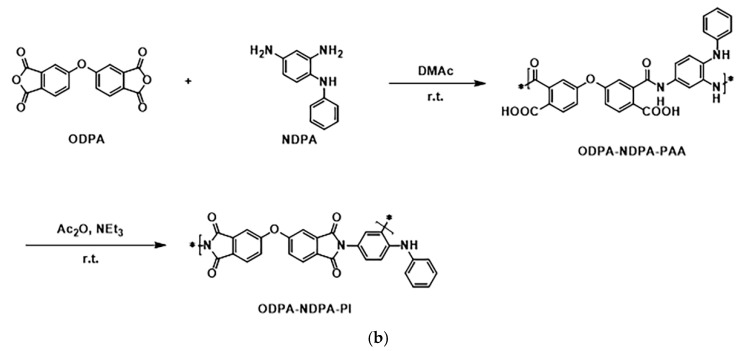
(**a**) Synthetic Routes of 2,4-diaminodiphenylamine monomer and (**b**) ODPA-NPDA-PI.

As shown in Figure 1a, the NPDA diamine monomer was synthesized in two steps, with successful synthesis confirmed by ^1^H NMR (Figure S1, Supporting Information) and mass spectrometry (Figure S2, Supporting Information). FTIR analysis (Figure S3, Supporting Information) verified NPDA’s structure, showing a broad absorption envelope at 3300–2500 cm^−1^ (centered at 3420 cm^−1^) characteristic of overlapping N-H stretches from primary (-NH_2_) and secondary (-NH-) amines, indicative of multiple polar amino groups with strong hydrogen bonding. Additional evidence includes aromatic C-H stretching at 3060 cm^−1^ and C=C vibration at ~1500 cm^−1^ confirming the benzene framework, while the strong ~1280 cm^−1^ peak corresponds to aromatic C-N bonds. Diagnostic out-of-plane C-H bending at ~810 cm^−1^ and ~895 cm^−1^, respectively, confirm 1,2,4-trisubstituted and monosubstituted benzene rings, consistent with 2,4-diaminodiphenylamine. Mass spectrometry (ESI) definitively confirmed the molecular structure through the protonated molecular ion at *m*/*z* = 199.9 [M+H]^+^, matching the calculated mass of 199.11 for C_12_H_13_N_3_. The 1H-NMR of the intermediate product C is shown in Figure S6 (Supporting Information).

### 3.2. Synthesis and Characterization of PI

**Figure 2:**
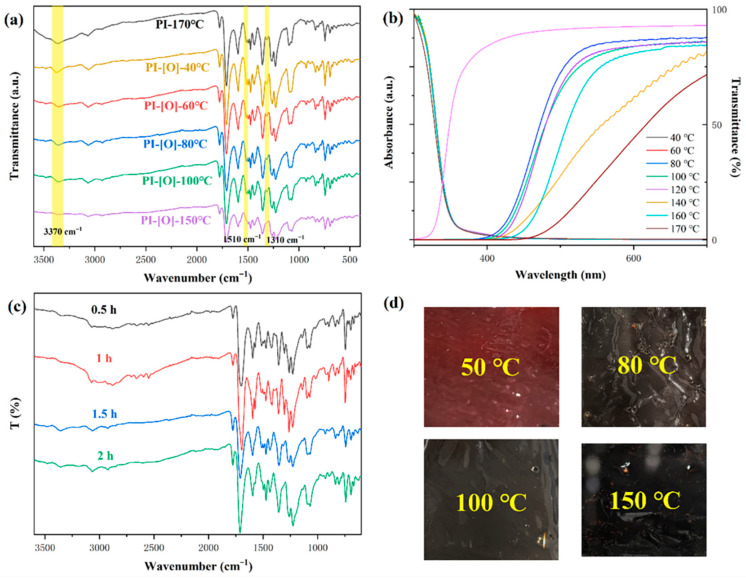
(**a**) FTIR spectra of PI films with different reaction temperature. (**b**) Transmission and absorption UV-vis spectra for PI (0.07 mg/mL in DMAc) with different reaction temperature. (**c**) FTIR spectra of PI films with different reaction time at 150 °C. (**d**) The photo images of PI films.

ODPA-NPDA-PI was synthesized via a conventional two-step method (Figure 1b). The process involves reacting NPDA with ODPA to form poly (amic acid) (PAA), the Mw of ODPA-NPDA-PAA is 12.11 × 10^4^ g/mol, the PDI (Table S1, Supporting Information) is 1.95 which indicates that the dispersion coefficient of ODPA-PAA-PAA is relatively low, and the molecular weight distribution is relatively narrow. Followed by chemical imidization to yield the polyimide. Successful imidization was confirmed by FTIR spectroscopy (Figure 2a), with characteristic absorption bands at 1778 and 1734 cm^−1^ (asymmetric and symmetric C=O stretching), 1373 cm^−1^ (C-N stretching), and 3370 cm^−1^ (N-H stretching) [32,33,34]. The successful synthesis of the polymer was further confirmed by ^1^H NMR spectroscopy (Figure S4, Supporting Information). These spectral features verify the formation of ODPA-NPDA-PI. The molecular weight of TPCPOHPPI was characterized via GPC. The weight average molecular weight (Mw) and polydispersity (PDI) of ODPA-NPDA-PAA were 12.11 × 10^4^ and 1.95, respectively.

### 3.3. Effect of Reaction Conditions on Optical Properties

We prepared films by mixing ODPA-NPDA-PI resin with mCPBA at room temperature, followed by coating onto a glass plate. During film formation, we observed that increasing the temperature or extending the reaction time systematically changed the system color from bright red to brown-red, ultimately turning near-black at elevated temperatures (Figure 2d). To investigate this phenomenon, we conducted systematic analyses. FTIR spectra from temperature gradient experiments (Figure 2a) reveal that as temperature increases, the secondary amine N-H vibration peak at 3370 cm^−1^ disappears, while absorption peaks at 1510 cm^−1^ (C-C vibration) and 1310 cm^−1^ (C-N vibration) shift. Complementary FTIR results from reaction time gradients at 150 °C (Figure 2c) indicate that prolonged reaction time moderately increases oxidation degree under these harsh conditions. Furthermore, absorption and transmission spectra (Figure 2b) demonstrate that while conjugated system expansion causes only a slight redshift in dilute solution absorption, it significantly extends the optical cutoff wavelength of the bulk film. These systematic changes originate from the oxidation mechanism: at 150 °C, mCPBA completely oxidizes the secondary amine sites in the polymer chain, yielding an imine structure. This enhances conjugation, which not only alters the vibration frequencies of adjacent C-N and C-C bonds (manifested as FTIR peak shifts) but also extends the overall conjugated system, thereby accounting for the color transitions and alterations in optical properties [23].

### 3.4. Oxidation-Dependent Optical Properties of PI

**Figure 3:**
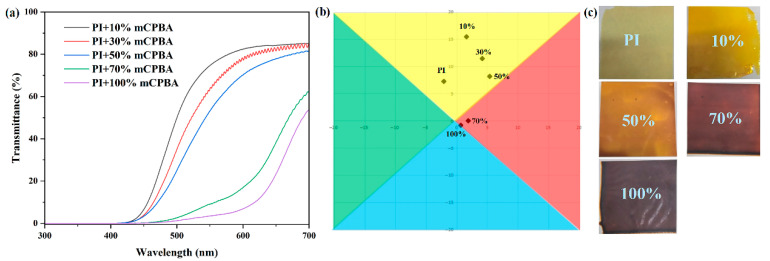
(**a**) UV/vis transmittance spectra, (**b**) CIELAB, and (**c**) a scanned photo of PI-x%.

Notably, by controlling the oxidant loading, we achieved tunable oxidation levels in the PI films, resulting in distinct color variations. As shown in Figure 3a, increasing the molar ratio of oxidant to diamine units (NPDA) from 10% to 100% progressively enhanced the oxidation degree. FTIR analysis confirmed the concomitant expansion of the intramolecular conjugated system. This structural modification significantly strengthens intramolecular charge transfer (CT) interactions, reducing the optical absorption gap [26]. Consequently, the optical cutoff wavelength exhibits a pronounced redshift, reaching 592 nm at a 1:1 oxidant/NPDA ratio. At this oxidation level, the film shows 5.9% transmittance at 600 nm with an average visible-light transmittance (400–700 nm) of 5.87%.

The CIE-Lab color system is widely employed for color characterization [29]. In this system, the L* value represents lightness (ranging from 0 for black to 100 for white), while the a* and b* coordinates correspond to the red-green and yellow-blue color components, respectively. Chroma ($C^{*}=\sqrt{\left(a^{2}+b^{2}\right)}$) quantifies color saturation [35]. Lower L* and C* values typically indicate a color closer to pure black. As presented in Table 1, the fully oxidized PI film exhibits a remarkably low C value of 1.09. This indicates near non-selective absorption across the visible spectrum, characteristic of an achromatic body. Its L* value of 29.94 corresponds to a dark gray appearance.

Critically, for materials approaching achromatic behavior, characterized by nearly uniform, non-selective absorption throughout the visible range, the overall average transmittance (T_avg_) is the primary physical determinant of the lightness index L*. Since achromatic bodies lack selective wavelength absorption, their perceived “color” is essentially a shade of gray. Consequently, the L* value directly correlates with the total transmitted luminous flux: lower transmittance yields a smaller L*, resulting in a darker material. Therefore, in this study, achieving an extremely low average visible-light transmittance (400–700 nm) of 5.87% through oxidative control is the underlying cause of the dark gray film with L* = 29.94.

This study presents a distinct and practical in situ oxidation strategy utilizing commercial ODPA/NPDA monomers and established polymerization to prepare precursors, followed by controlled post-processing oxidation with mCPBA (adjusting temperature/time/dosage) for tunable coloration. This approach bypasses complex monomer design while achieving functionally dark-gray films (L* = 29.94, C* ≈ 1.09, λ_cut_ = 592 nm) with ultralow visible transmittance (~5.87%), meeting core optical requirements for black PI films.

### 3.5. Mechanistic Investigation of mCPBA Oxidation

**Figure 4:**
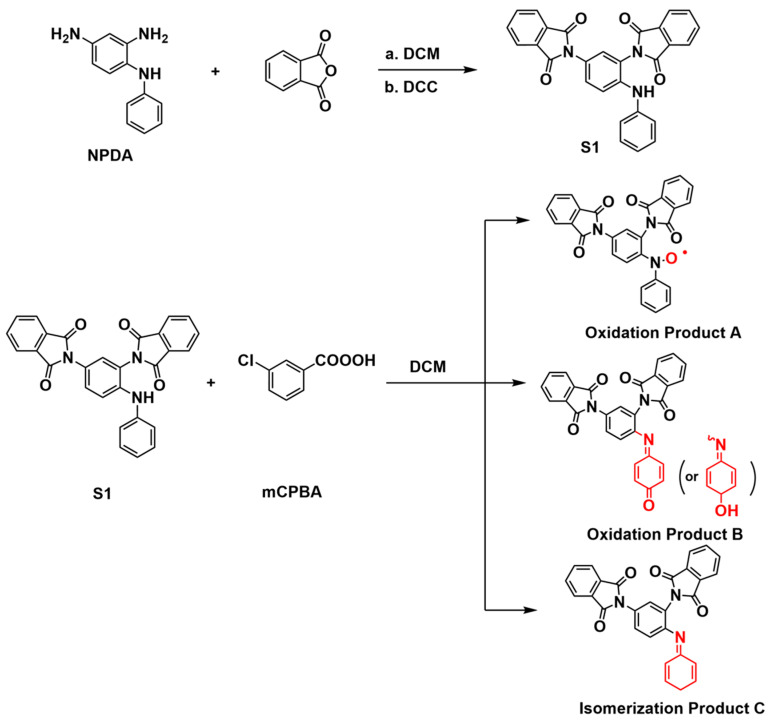
Proposed Structures of Oxidation Products.

Leveraging the inherent high reducibility of NPDA, we developed a strategy for precise color control in PI films by in situ oxidation to modulate their conjugated structure. To elucidate the molecular mechanism underlying this color change which is attributed to oxidative structural transformations causing the observed absorption redshift (see supporting evidence in Figure 4), we designed and conducted structural characterization experiments on isolated oxidation products of the diamine monomer.

**Figure 5:**
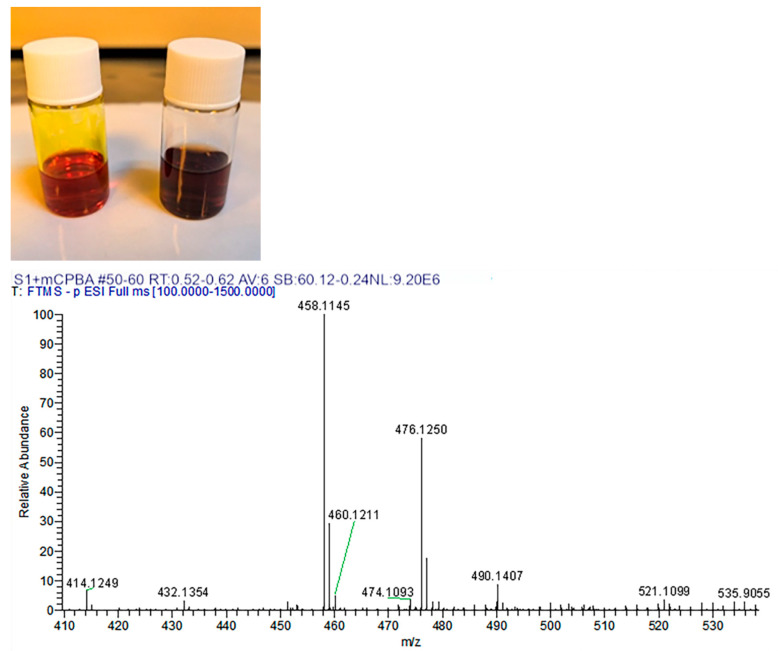
Color Evolution and Post-Oxidation Mass Spectrometry Analysis via In Situ Oxidant Addition.

Reaction of the protected intermediate S1 (structure fully confirmed by ^1^H NMR, see Figure S5, supporting information) with 3-chloroperoxybenzoic acid (mCPBA) yielded a complex mixture of oxidation products, as revealed by high-resolution mass spectrometry (HRMS). The mass spectrometric fragment data of S1-derived oxidation products are presented in Table 2. Comprehensive characterization indicates that mCPBA oxidation primarily targets the unprotected secondary amine (-NH-) site in S1. The core chemical transformation involves oxidation of the nitrogen lone pair, converting the -NH- moiety into an imine (-N=) or quinoid-like structure (key structural changes illustrated in Figure 5) with enhanced electron-withdrawing capacity and extended conjugation. This increased conjugation and the introduction of new chromophores reduce the energy gap between the highest occupied molecular orbital (HOMO) and the lowest unoccupied molecular orbital (LUMO), significantly narrowing the optical absorption gap [30]. Consequently, the molecule absorbs lower-energy photons at longer wavelengths, resulting in a red-shifted and broadened absorption spectrum that extends into the red region [36]. This electronic transition directly transforms the color from the bright red of intermediate S1 to the dark brown observed in the oxidized mixture.

### 3.6. Thermal Properties and Mechanical Properties of PI

Thermal stability and glass transition temperature (T_g_) of the intrinsically black polyimide films were characterized by thermomechanical analysis (TMA) (Figure S7b, supporting information) and dynamic mechanical analysis (DMA) (Figure S7a, supporting information). The T_g_ values, determined from the peak temperature of tan δ in DMA curves, along with all thermal performance data are summarized in Table 3. Coefficient of thermal expansion (CTE) was measured via TMA over 50–250 °C. The T_5%_ (Figure S8, supporting information) values of the PI films, including PI-30%, PI-50%, PI-70% and PI-100%, at which 5% weight loss occurred, exceed 309 °C (Table 3). Compared to pure PI, the initial decomposition temperature of PI-x decreased due to the instability caused by the oxidation of the NPDA side chains.

As summarized in Table 3, the intrinsically black polyimide films produced via this method exhibit robust mechanical properties: a tensile strength of 124 MPa and an initial modulus of 3.2 GPa. The mechanical performance of polyimide films is influenced by multiple factors—including chemical structure, molecular weight, and processing conditions—with structure being the most significant determinant. Following in situ oxidation, structural modifications occur in the diamine moiety, leading to a reduction in elongation at break. Nevertheless, the films retain substantial tensile strength and initial modulus. Consequently, these intrinsically black polyimide films maintain mechanical properties fully adequate for photolithography mask applications.

## 4. Conclusions

This work successfully synthesized a novel diamine monomer featuring pendant diphenylamine groups and its corresponding soluble polyimide. Capitalizing on the monomer’s high reduction susceptibility, in situ oxidation was implemented during polyimide film formation using oxidizing agents, enabling precise color modulation through controlled oxidation levels, which culminating in the controllable preparation of near-black films. This strategy seamlessly integrates into conventional processing workflows without protocol modifications, while its inherent simplicity, operational ease, and cost-effectiveness render it readily extensible to other reduction-susceptible monomers, demonstrating significant application potential. Critically, optimized sequential chemical imidization/oxidation prevented imidization interference and preserved mechanical properties. The strategy’s simplicity and scalability, eliminating difficult monomer synthesis, provide a viable route to produce dark/black PI optical films from mature monomer systems via straightforward post-processing, offering significant promise for cost-sensitive applications like light-blocking layers in flexible electronics. Future research could prioritize optimizing oxidation systems, particularly developing more efficient, milder, and lower-cost oxidants, achieving deeper black coloration (L < 20) via precise oxidation control, and systematically evaluating long-term stability alongside critical properties like electrical insulation and thermal stability to fully unlock the technology’s potential.

## Figures and Tables

**Table 1:** Optical Properties of the PIs.

PI *^a^*	λ_cut_ *^b^* (nm)	T_600_ *^c^* (%)	T_avg_ *^c^* (%)	L* *^d^*	a* *^d^*	b* *^d^*	c* *^d^*	Reference
PI-0	419	86.5	82.91	41.03	−2.07	7.22	7.51	this work
PI-10%	446	82.4	72.61	40.01	1.63	15.42	15.5	this work
PI-30%	454	77.7	63.09	38.98	4.21	11.41	12.2	this work
PI-50%	456	70.2	46.43	36.71	5.42	8.14	9.78	this work
PI-70%	520	16.9	12.67	30.31	1.92	−0.01	1.92	this work
PI-100%	591	5.9	5.87	29.94	0.70	−0.84	1.09	this work
PI-a	-	0.0	-	20.8	0.8	−2.6	2.72	[26]

*^a^* The thickness of the PI sample is 50 μm. *^b^* λ_cut_: Cutoff wavelength. *^c^* T_600_: Transmittance at the wavelength of 600 nm. T_avg_: The average transmittance in the range of 300–700 nm. *^d^* L*, a*, b*, C*: CIE Lab optical parameters.

**Table 2:** Mass spectrometric fragmentation data for S1-derived oxidation products.

*m*/*z* (Measured Value)	Fragment Assignment	Molecular Formula	*m*/*z* (Theoretical Value)	Fragmentation Pathway
476.1250	[M]^+•^	C_28_H_18_N_3_O_5_	476.1246	Molecular Ion Peak
458.1145	[M-H_2_O]^+•^	C_28_H_16_N_3_O_4_	458.1140	Dehydration
432.1354	[M-CO_2_]^+^	C_27_H_18_N_3_O_3_	432.1348	Decarboxylation
414.1249	[M-CO_2_-H_2_O]^+•^	C_27_H_16_N_3_O_2_	414.1242	Dehydration
474.1093	[M-H_2_]^+•^	C_28_H_16_N_3_O_5_	474.1090	Dehydrogenation

**Table 3:** Thermal Properties and Mechanical Properties of PI.

PI	T_g_ *^a^* (°C)	T_5%_ (°C)	CTE (ppm/K)	σ *^b^* (Mpa)	E *^c^* (Gpa)	ε_b_ *^d^* (%)
PI	321.6	395.4	33	151 ± 4	2.4 ± 0.2	21 ± 1
PI-30%	324.1	375.5	35	128 ± 1	2.1 ± 0.2	17 ± 1
PI-50%	323.8	374.8	34	142 ± 4	2.6 ± 0.1	12 ± 1
PI-70%	330.2	375.2	33	122 ± 2	2.5 ± 0.1	10 ± 1
PI-100%	332.5	372.9	36	124 ± 4	3.1 ± 0.1	8.7 ± 1

*^a^* T_g_: glass transition temperature tested by DMA. *^b^* σ: tensile strength. *^c^* E: initial modulus. *^d^* ε_b_: elongation at break.

## Data Availability

The original contributions presented in this study are included in the article and supplementary material. Further inquiries can be directed to the corresponding authors.

## Supplementary Materials

The following supporting information can be downloaded at: https://www.mdpi.com/article/10.3390/polym17212876/s1. Table S1. Molecular weights of ODPA-NPDA-PAA. Figure S1. The 1H-NMR spectra of NPDA (DMSO-d6). Figure S2. MS spectrum of NPDA. Figure S3. IR spectra of NPDA. Figure S4. The 1H-NMR spectra of ODPA-NPDA-PI (DMSO-d6). Figure S5. The 1H-NMR spectra of S1 (DMSO-d6). Figure S6. The 1H-NMR spectra of C (DMSO-d6). Figure S7. DMA (a) and TMA (b) curves of the PI-[O] film. Figure S8. TGA curves of the PI and PI-[O] film.
