# Supplementary material for: Color-Tunable Intrinsically Black Polyimides: A Facile Strategy via In Situ Oxidation Color Control
Source: Polymers (Basel). 2025 Oct 28;17(21):2876. doi: 10.3390/polym17212876 (PMC12609245; doi:10.3390/polym17212876)
Supplement: Supplementary file 1 [file polymers-17-02876-s001.zip › polymers-3922665-supplementary.pdf]

Supporting information

Color-Tunable Intrinsically Black Polyimides: A Facile Strategy via *In Situ* Oxidation Color Control

Desheng Kong

Chang Chun Institute of Applied Chemistry Chinese Academy of Sciences Changchun, China CHINA

Jiaojiao Ma*

Chang Chun Institute of Applied Chemistry Chinese Academy of Sciences Changchun, China CHINA

Zeyu Li

Chang Chun Institute of Applied Chemistry Chinese Academy of Sciences Changchun, China CHINA

Shun Shi

Chang Chun Institute of Applied Chemistry Chinese Academy of Sciences Changchun, China CHINA

Tong Yuan

Chang Chun Institute of Applied Chemistry Chinese Academy of Sciences Changchun, China CHINA

Jianfeng Qian

Chang Chun Institute of Applied Chemistry Chinese Academy of Sciences Changchun, China CHINA

Haiquan Guo*

Chang Chun Institute of Applied Chemistry Chinese Academy of Sciences Changchun, China CHINA

This document is the results of the research project funded by the National Science Foundation.

*Corresponding author

Email addresses: hqguo@ciac.ac.cn (Haiquan Guo), jjma@ciac.ac.cn (Jiaojiao Ma)

1.Address: No. 5625, Renmin Avenue, Changchun City, Jilin Province, China, 130022

2.Address: School of Applied Chemistry and Engineering, University of Science and Technology of China, Hefei, Anhui, China 230026

List of charts

Table S1. Molecular weights of ODPA-NPDA-PAA. 1

Fig S1. The 1H-NMR spectra of NPDA (DMSO-d6). 2

Fig S2. MS spectrum of NPDA. 2

Fig S3. IR spectra of NPDA. 3

Fig S4. The 1H-NMR spectra of ODPA-NPDA-PI (DMSO-d6). 3

Fig S5. The 1H-NMR spectra of S1 (DMSO-d6). 4

Fig S6. The 1H-NMR spectra of C (DMSO-d6). 4

Fig S7. DMA (a) and TMA (b) curves of the PI-[O] film. 5

Fig S8. TGA curves of the PI and PI-[O] film. 5

Table S1. Molecular weights of ODPA-NPDA-PAA.

Sample	Mn[a] ($\times 10^4$ g/mol)	Mw[a] ($\times 10^4$ g/mol)	PDI
ODPA-NPDA-PAA	6.21	12.11	1.95

[a] Mn: number average molecular weight; Mw: weight average molecular weight; PDI: polydispersity index, PDI= Mw/Mn.

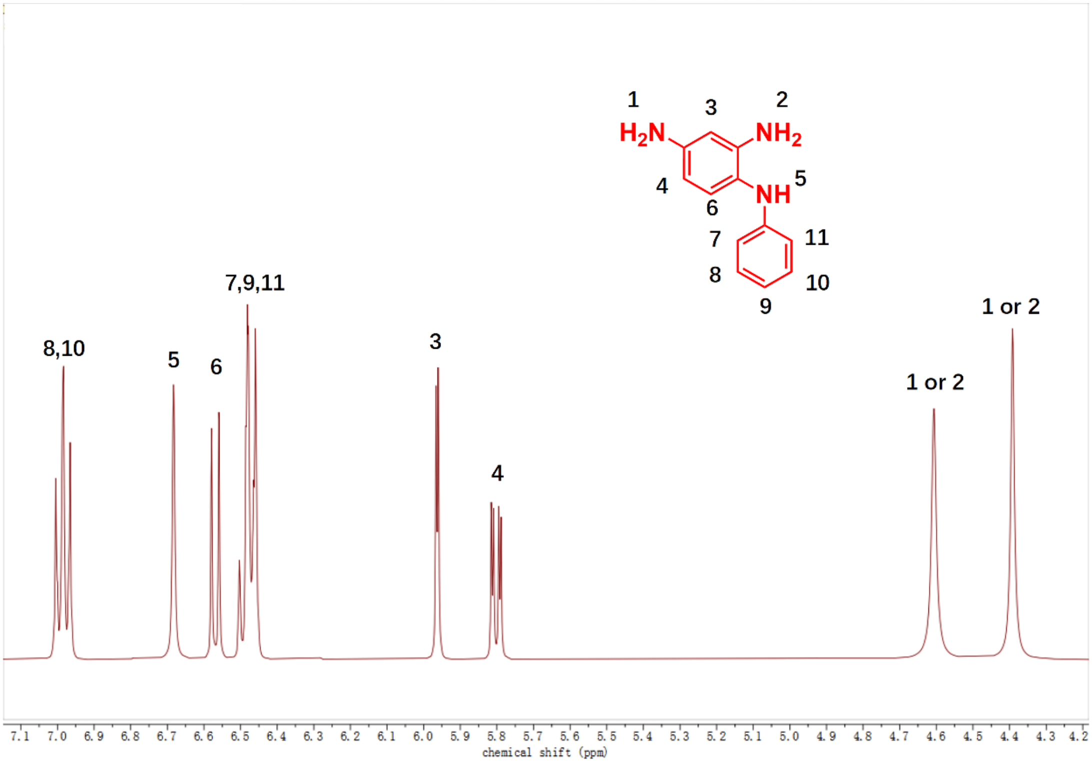

Fig S1. The 1H-NMR spectra of NPDA (DMSO-d6).

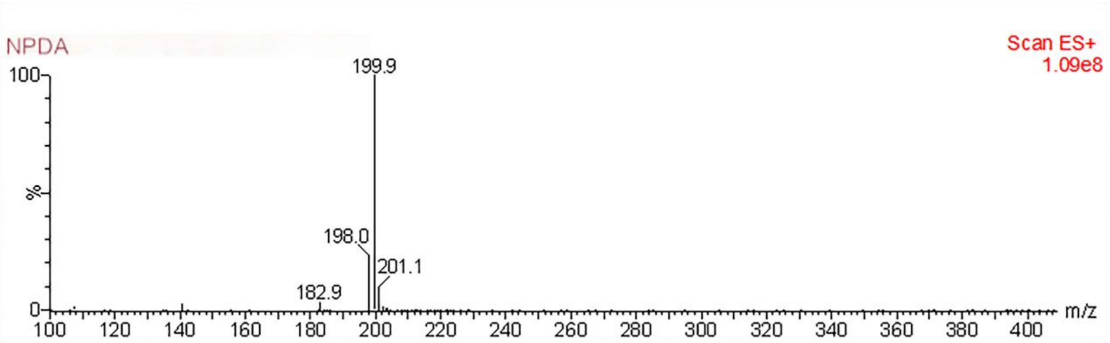

Fig S2. MS spectrum of NPDA.

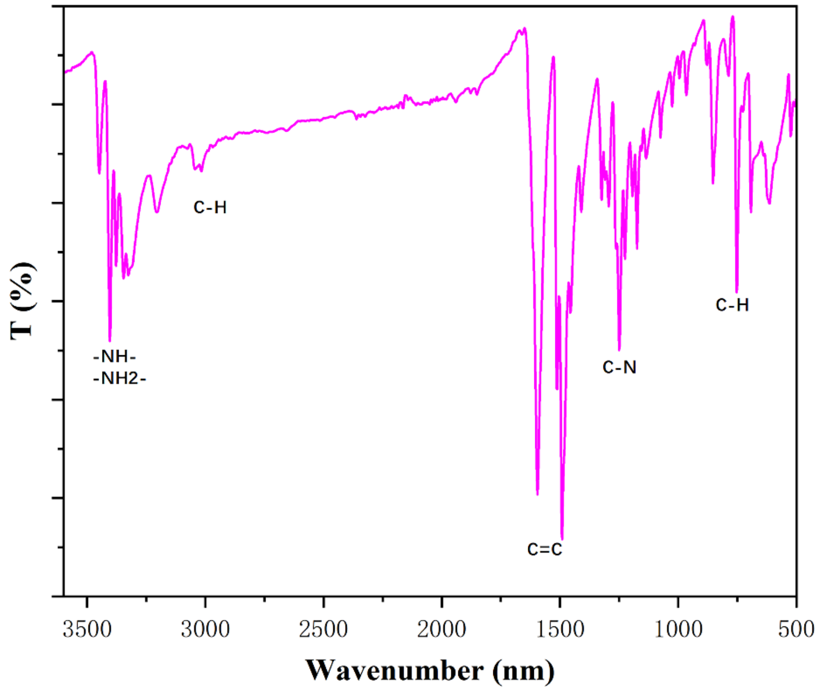

Fig S3. IR spectra of NPDA.

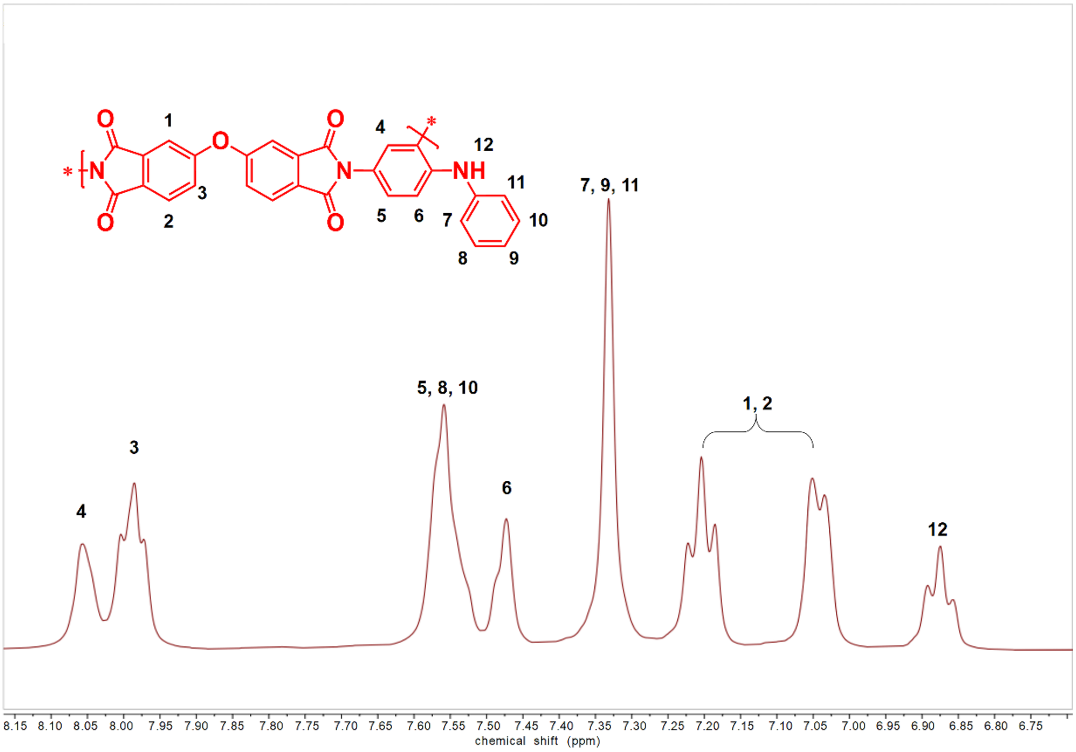

Fig S4. The 1H-NMR spectra of ODPA-NPDA-PI (DMSO-d6).

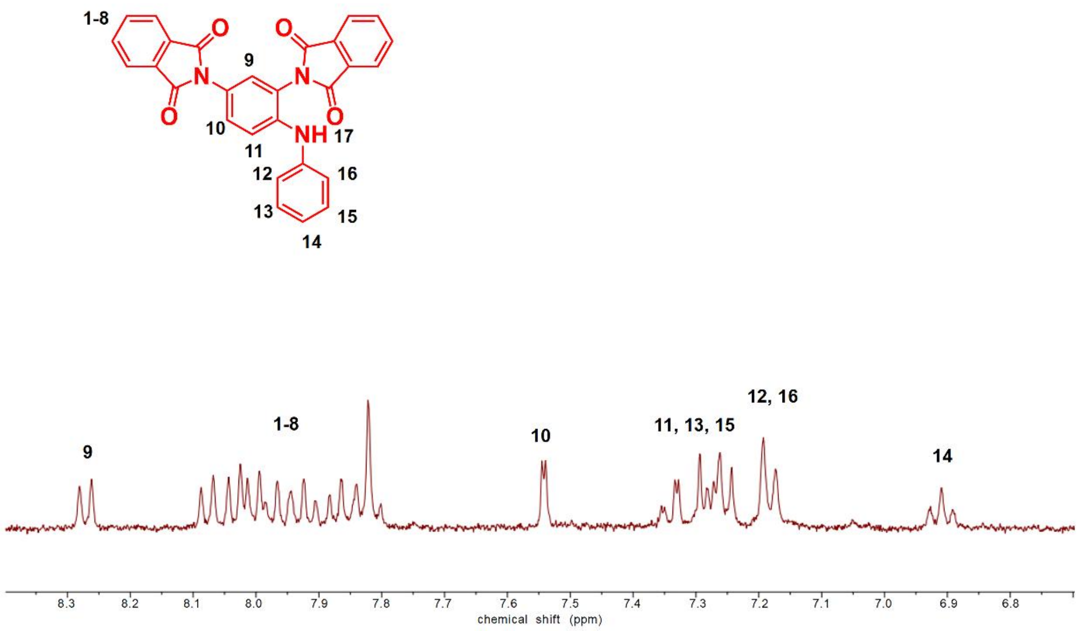

Fig S5. The 1H-NMR spectra of S1 (DMSO-d6).

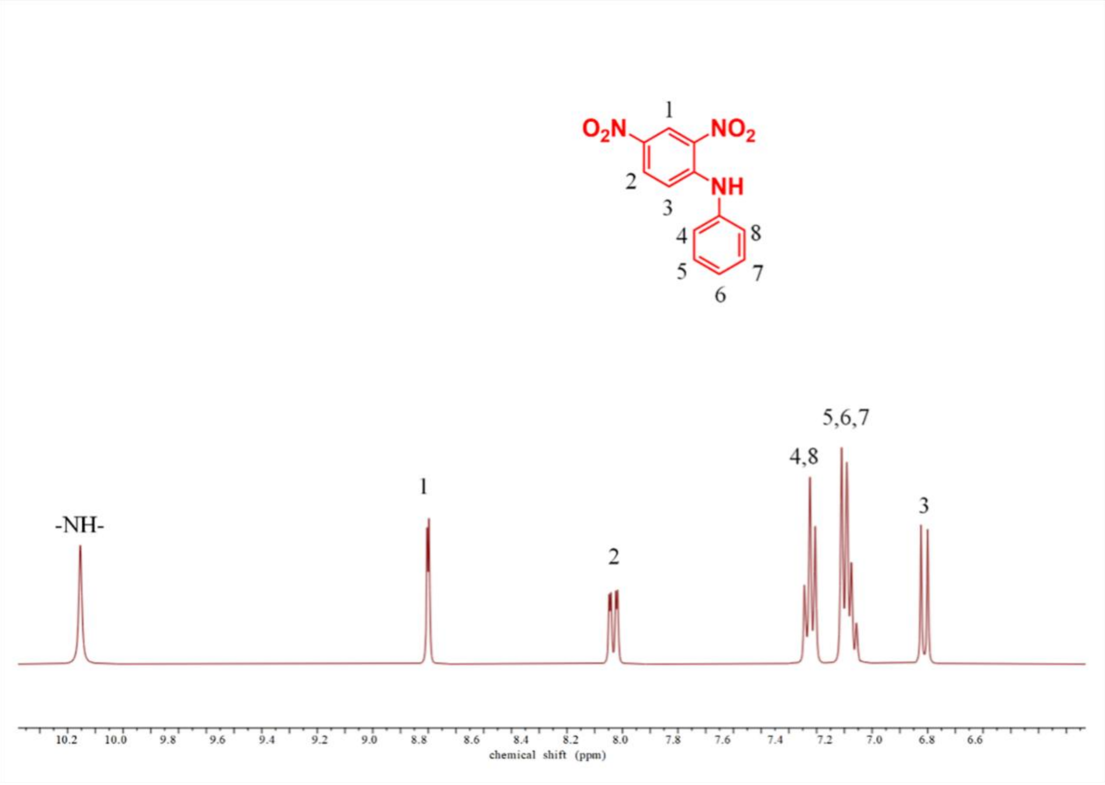

Fig S6. The 1H-NMR spectra of C (DMSO-d6).

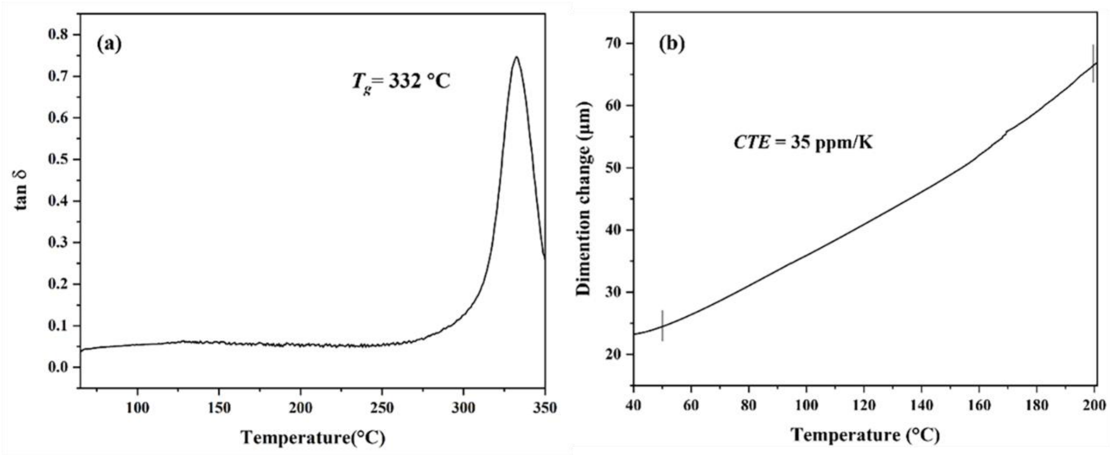

Fig S7. DMA (a) and TMA (b) curves of the PI-[O] film.

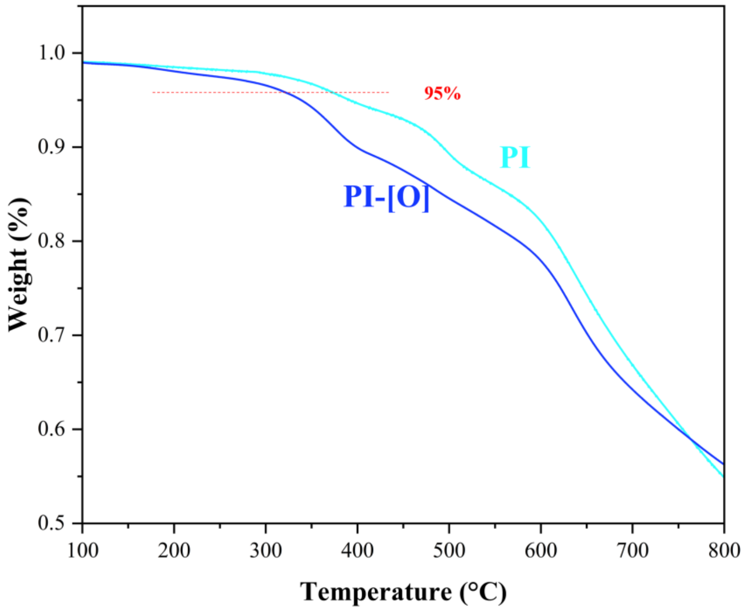

Fig S8. TGA curves of the PI and PI-[O] film.
